# Antitumour effect of cyclin-dependent kinase inhibitors (p16^INK4A^, p18^INK4C^, p19^INK4D^, p21^WAF1/CIP1^ and p27^KIP1^) on malignant glioma cells

**DOI:** 10.1038/sj.bjc.6600862

**Published:** 2003-04-15

**Authors:** T Komata, T Kanzawa, H Takeuchi, I M Germano, M Schreiber, Y Kondo, S Kondo

**Affiliations:** 1 Department of Neurosurgery, Mount Sinai School of Medicine, New York, NY 10029, USA; 2 Department of Neurosurgery, The University of Texas M.D. Anderson Cancer Center, Houston, TX 77030, USA; 3 Department of Biology, McMaster University, Hamilton, Ont. Canada L8S 4K1

**Keywords:** cyclin-dependent kinase inhibitors, p27^KIP1^, glioma, autophagy

## Abstract

Cyclin-dependent kinase inhibitors (CDKIs) are considered as novel anticancer agents because of their ability to induce growth arrest or apoptosis in tumour cells. It has not yet been fully determined, however, which CDKI is the best candidate for the treatment of malignant gliomas and whether normal brain tissues are affected by CDKI expression. Using recombinant adenoviral vectors that express CDKIs (p16^INK4A^, p18^INK4C^, p19^INK4D^, p21^WAF1/CIP1^ and p27^KIP1^), we compared the antitumour effect of CDKIs on malignant glioma cell lines (A172, GB-1, T98G, U87-MG, U251-MG and U373-MG). p27^KIP1^ showed higher ability to suppress the growth of all tumour cells tested than other CDKIs. Interestingly, overexpression of p27^KIP1^ induced autophagic cell death, but not apoptosis in tumour cells. On the other hand, p27^KIP1^ overexpression did not inhibit the viability of cultured astrocytes (RNB) nor induced autophagy. Overall, our findings suggest that gene transfer of p27^KIP1^ may be a promising approach for the therapy of malignant gliomas.

Cell cycle progression is controlled by cyclin-dependent kinases (CDKs) that are activated by cyclin binding (Sherr, 1994; Morgan, 1995) and inhibited by CDK inhibitors (CDKIs) (Reed *et al*, 1994; Sherr and Roberts, 1999). Two classes of CDKIs negatively regulate the cell cycle (Sherr and Roberts, 1995; Harper and Elledge, 1996; Xiong, 1996). One class, the inhibitor of CDK4 (INK4) family, which consists of p15^INK4B^, p16^INK4A^, p18^1NK4C^ and p19^INK4D^, specifically binds CDK4 or CDK6 and inhibits cyclin D association (Serrano *et al*, 1993; Guan *et al*, 1994; Hannon and Beach, 1994; Hirai *et al*, 1995). The other class of CDKIs, the kinase inhibitor protein (KIP) family, p21^WAF1/CIP1^, p27^KIP1^ and p57^KIP2^, binds and inhibits cyclin-bound CDKs (Harper *et al*, 1993; Toyoshima and Hunter, 1994; Lee *et al*, 1995).

Recent investigations show that exogenous induction of CDKIs induces growth arrest or apoptosis in tumour cells, indicating the potential use of CDKIs as a therapeutic tool (Guan *et al*, 1994; Craig *et al*, 1997; Hiromura *et al*, 1999). However, it has not been fully investigated which CDKI is the most useful for cancer therapy and whether normal tissues are affected by CDKI expression. To address these issues, in the present study, we compared the antitumour effect of CDKIs on malignant glioma cell lines and cultured astrocytes by using recombinant adenoviral vectors that express CDKIs (p16^INK4A^, p18^INK4C^, p19^INK4D^, p21^WAF1/CIP1^ and p27^KIP1^).

## MATERIALS AND METHODS

### Cells

Malignant glioma cells (A172, GB-1, T98G, U87-MG, U251-MG and U373-MG) (Komata *et al*, 2000) and cultured astrocytes RNB (Kondo *et al*, 1996) were cultured in Dulbecco's modified Eagle's medium (DMEM, GIBCO BRL, Grand Island, NY, USA) supplemented with 10% fetal bovine serum (GIBCO BRL), 4 mM glutamine, 100 U ml^−1^ penicillin and 100 *μ*g ml^−1^ streptomycin.

### Construction of recombinant adenoviral vectors

The recombinant AdMH4p16, AdMHp18, AdMH4p19, AdMH4p21 and AdMH4p27 containing p16^INK4A^, p18^INK4C^, p19^INK4D^, p21^WAF1/CIP1^ and p27^KIP1^ were kindly supplied from Dr FL Graham (McMaster University, Ontario, Canada). As described previously (Schreiber *et al*, 1999), human p16 cDNA (pBluescriptSK-p16) was obtained from Dr D Beach (Cold Spring Harbor, New York, USA). pxep21, pAdcp17 and pAdcp19 were obtained from Dr T Thompson (Baylor College of Medicine, Houston, TX, USA), and pSCZhuwtp27 was a gift from Dr J Roberts (Fred Hutchison Cancer Research Center, Seattle, WA, USA). AdBHGΔ1,3 containing El and E3 deletions is a control recombinant adenovirus that has identical backbone sequences to the adenoviral constructs expressing CDKIs.

### Adenoviral infection

The effect of CDKIs on cell viability was determined by using a trypan blue dye exclusion assay as described previously (Komata *et al*, 2000). To achieve the infectivity of >90%, A172, GB-1, U87-MG, U251-MG, U373-MG and RNB cells were tested at a multiplicity of infection (MOI) of 60 PFU cell^−1^. On the other hand, T98G cells were infected with 180 MOI. The percentage of cell viability was calculated from the mean cell viability of treated cells divided by that of cells with control treatment. To detect the expression of CDKI in infected cells, the immunoblotting assays using anti-CDKI antibody (Santa Cruz Biotechnology, Santa Cruz, CA, USA) were performed as described previously (Kondo *et al*, 1996).

### Detection of apoptotic or autophagic cell death

To detect apoptosis, the terminal deoxynucleotidyl transferase (Tdt)-mediated nick end labelling (TUNEL) analysis was performed as described previously (Komata *et al*, 2000). To detect autophagic changes in infected cells, cells were stained with acridine orange (Poly-sciences, Warrington, PA, USA) as described previously (Paglin *et al*, 2001). At 2 days after adenoviral infection, acridine orange was added at a final concentration of 1 *μ*g ml^−1^ for 15 min. Microphotographs were obtained with a fluorescence microscope.

### Statistical analysis

The data were expressed as means ±s.d. Statistical analysis was performed by using Student's *t*-test (two-tailed). The criterion for statistical significance was taken as *P*<0.05.

## RESULTS

### Effect of CDKI overexpression on viability of malignant glioma cells

**Figure 1:**
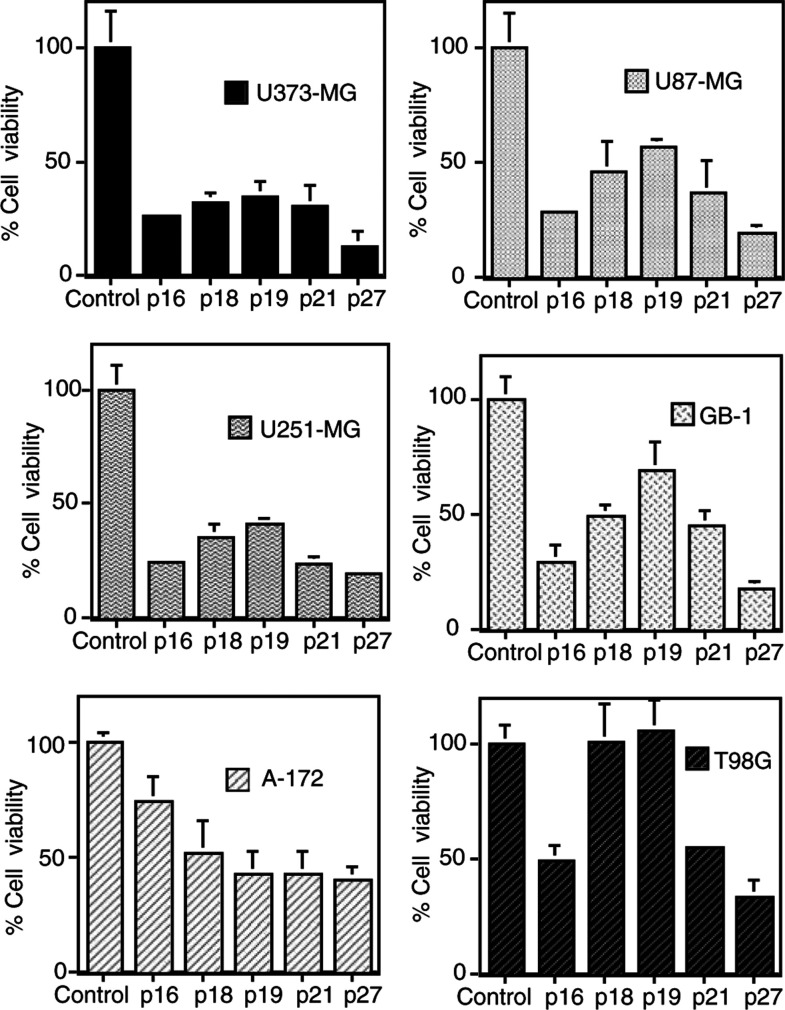
Effect of adenovirus expressing CDKIs on cell viability of malignant glioma cell lines. Tumour cells were seeded at 5 × 10^3^ cells well^−1^ (0.1 ml) in 96-well flat-bottomed plates and incubated overnight at 37°C. On the following day, U373-MG, U251-MG, U87-MG cells, A172 cells, GB-1 and T98G cells were infected with AdBHGΔl,3, AdMH4pl6, AdMH4pl8, AdMH4pl9, AdMH4p21 or AdMH4p27 as indicated (day 0). On day 3, the cell viability was determined using a trypan blue dye exclusion assay. Values are given as the percentage of viable cells of AdBHGΔl ,3-infected cultures. Results shown are the means±s.d. of three independent experiments. U373-MG, U251-MG, U87-MG, A172 and GB-1 cells were infected at 60 MOI, while 180 MOI was used for T98G cells.

To investigate the effect of CDKI on malignant glioma cell lines, cell viability was determined 3 or 5 days after adenoviral infection. As shown in Figure 1

, the treatment of U373-MG cells with AdMH4p16^INK4A^, AdMH4p18^INK4C^, AdMH4p19^INK4D^, AdMH4p21^WAF1/CIP1^ or AdMH4p27^KIP1^ for 3 days significantly inhibited the cell viability compared to that with control vector (*P*<0.002 to *P*<0.004). In U251-MG, U87-MG, A172 and GB-1, similar antitumour effects were observed with each CDKI 3 days after adenoviral infection (*P*<0.0002 to *P*<0.02). On the other hand, the effect of AdMH4p18^INK4C^ or AdMH4p19^INK4D^ was not significant for T98G cells (*P*=0.17 or *P*=0.71), although other CDKIs were effective (*P*<0.0001 to *P*<0.03). Among the CDKIs used in the present study, p27^KIP1^ was more effective for tumour cells than the other CDKIs. The number of viable cells of U373-MG treated with AdMH4p27^KIP1^ decreased below the initial cell number (5000): 4138±1794 (day 3) or 2555±1042 (day 5). This indicates that approximately half of p27^KIP1^-infected U373-MG cells underwent cell death by day 5. Additionally, cell number of other tumour cell lines decreased by 30–60% from the initial cell number like U373-MG cells 5 days after the treatment with AdMH4p27^KIP1^. These results indicate that 27^KIP1^ has the highest antitumour effect on all malignant glioma cells tested in the present study.

### Effect of p27^KIP1^ in RNB cells

**Figure 2:**
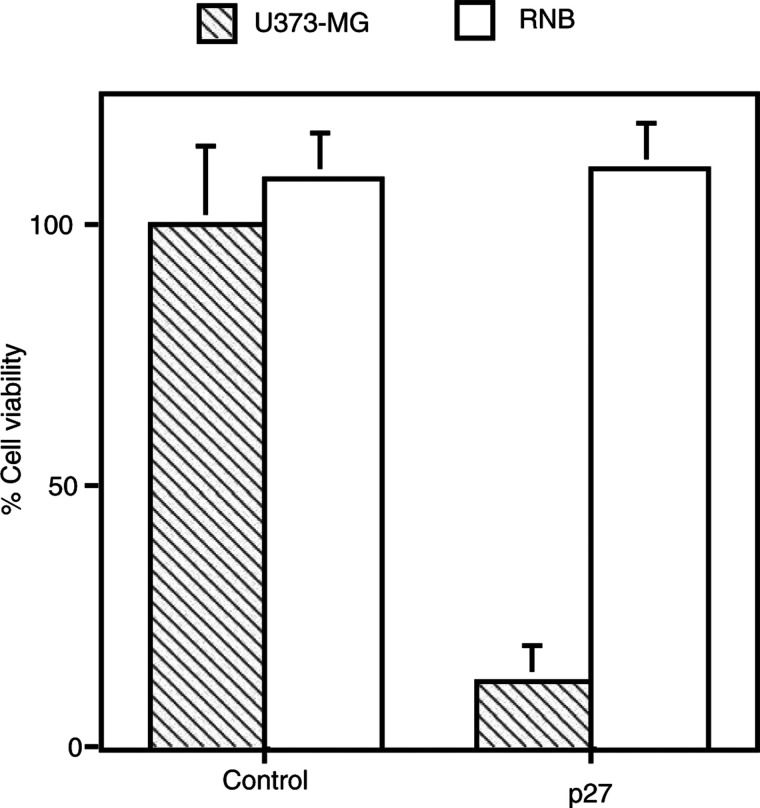
Effect of AdMH4p27 on RNB cells. U373-MG or RNB cells were seeded at 5 × 10^3^ cells well^−1^ (0.1 ml) in 96-well flat-bottomed plates and incubated overnight at 37°C. On the following day, cells were infected with AdBHGΔl,3 or AdMH4p27 at 60 MOI. On day 3, the cell viability was determined using a trypan blue dye exclusion assay. Values are given as the percentage of viable cells of AdBHGΔl,3-infected cultures. Results shown are the means±s.d. of three independent experiments.

To investigate whether normal brain tissues are affected by p27^KIP1^ expression, cultured rat astrocyte, RNB cells, were treated with AdMH4p27^KIP1^. As shown in Figure 2

, the effect of p27^KIP1^ overexpression was not significant for RNB cells, while the viability of U373-MG cells decreased to 15% of the control (*P*<0.005). This result suggested that p27^KIP1^ -based therapy will be selective for tumour cells.

### Effect of p27^KIP1^ on induction of apoptosis or autophagy in U373-MG cells

**Figure 3:**
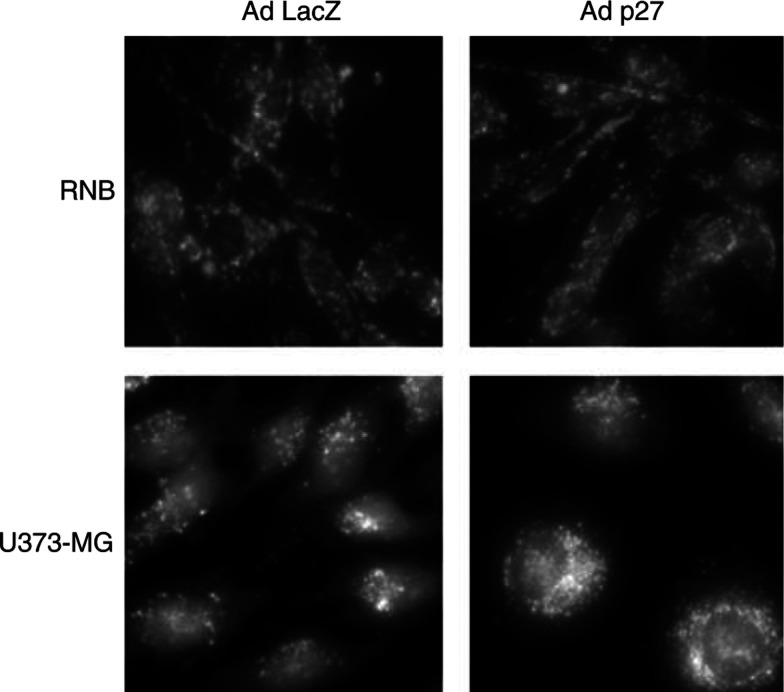
Effect of AdMH4p27 on induction of autophagy. At 72 h after the adenoviral infection with AdBHGΔl,3 or AdMH4p27, U373-MG or RNB cells were treated with 1 *μ*g ml^−1^ of acridine orange, and incubated at 37°C for 15 min. Viable cells were observed under the fluorescence microscope. Results shown are representative of three independent experiments.

To determine the type of cell death induced by AdMH4p27^KIP1^, we first performed the TUNEL staining. The incidence of TUNEL-positive cells in U373-MG cells treated with AdBHGΔ1,3 or AdMH4p27^KIP1^ for 3 or 5 days was less than 1%. Next, we investigated the changes in the cellular acidic compartments to detect the occurrence of autophagic cell death. Vital staining of U373-MG cells with acridine orange revealed the appearance of acidic vesicular organelles (AVO) 3 days after AdMH4p27^KIP1^ infection (Figure 3

). The staining of p27^KIP1^-infected cells clearly showed punctuate acidic vesicles that were diffusely distributed in cytoplasm. In contrast, there was no change of fluorescent signals in RNB cells treated with AdBHGΔ1,3 or AdMH4p27^KIP1^. These results indicated that the antitumour effect of p27^KIP1^ on malignant glioma cells was because of autophagic cell death.

## DISCUSSION

In the present study, we have demonstrated that p27^KIPl^ shows a greater antitumour effect than the other CDKIs (p16^INK4A^, p18^INK4C^, p19^INK4D^ or p21^WAF1/CIP1^) against six human malignant glioma cell lines. p27^KIPl^-infected tumour cells undergo autophagy, but not apoptotic cell death, while overexpression of p27^KIP1^ does not inhibit viability of cultured astrocytes. Our findings provide new insights into the potential use of p27^KIP1^ as a novel therapeutic tool.

p27^KIP1^ plays a central role in the negative control of cell growth. p27^KIPl^ is generally expressed at high levels in cells arrested by treatment with transforming growth factor-*β*, contact inhibition or serum deprivation (Koff *et al*, 1993; Polyak *et al*, 1994). In contrast, p27^KIP1^ declines in the presence of mitogenic growth factor signalling or interleukin-2 (Nourse *et al*, 1994; Cheng *et al*, 1998). p27^KIP1^-deficient mice develop a variety of abnormalities including multiorgan hyperplasia and pituitary tumours (Nakayama *et al*, 1996). Furthermore, the higher the levels of p27^KIP1^ expression, the better the prognosis with regard to human malignant gliomas (Alleyne *et al*, 1999), breast cancer (Porter *et al*, 1997) or lung cancer (Esposito *et al*, 1997). Therefore, introduction of p27^KIP1^ gene into tumour cells is expected to be a promising strategy to inhibit their malignant cellular proliferation.

It has been controversial whether p27^KIP1^ expression leads tumour cells to growth arrest or cell death. Some groups demonstrate the induction of growth arrest by p27^KIPl^ (Sherr and Roberts, 1995; Craig *et al*, 1997). Others show that p27^KIP1^ expression induces apoptosis in several cell lines (Katayose *et al*, 1997; Schreiber *et al*, 1999), while p27^KIPl^ protects cells from apoptosis (Hiromura *et al*, 1999). In the present study, p27^KIP1^ induced autophagic cell death, but not apoptosis in malignant glioma cells. Recently, several groups have proposed two types of programmed cell death (Schwartz *et al*, 1993; Bursch *et al*, 2000). Type I programmed cell death, or apoptosis, is mediated by caspases/bcl family, and has typical morphological and biochemical characteristics such as chromatin condensation or nucleosomal ladder formation. Since TUNEL-positive cells were not detected in p27^KIP1^-infected U373-MG cells in the present study, it is unlikely that apoptosis is involved in the antitumour effect of p27^KIP1^ on malignant glioma cells. In contrast, type II programmed cell death is marked morphologically by increased autophagy and early destruction of the cytoplasm that occur either without nuclear collapse or precedes it (Schwartz *et al*, 1993; Bursch *et al*, 2000). More recently, Paglin *et al* (2001) demonstrated that formation of acidic vesicles was detected in radiation-induced autophagy. Since the development of AVO was detected in p27^KIP1^-infected U373-MG cells, we suggest that the antitumour effect of p27^KIP1^ on malignant glioma cells is mainly because of induction of type II programmed cell death, autophagy. Further study is necessary to investigate the molecular mechanisms underlying p27^KIP1^-induced autophagy in malignant glioma cells.

In summary, p27^KIP1^ shows the most potent antitumour effect against malignant glioma cells, while cultured astrocytes are insensitive to p27^KIPl^ expression. The effect is because of autophagic cell death as well as G0/G1 growth arrest. Therefore, we expect that p27^KIP1^-based therapy for malignant gliomas might be a promising approach that is worth exploring further.
